# Efficient wet-spinning of pre-aligned microtissues for 3D bioprinting complex tissue alignment

**DOI:** 10.1088/1758-5090/add37f

**Published:** 2025-05-16

**Authors:** Caleb D Vogt, Joseph R Broomhead, Kyle Y Kunisaki, Johanna Margaret Teegarden, Kallie L Frett, Kyleigh Q Pacello, Anthony H Vitale, Angela Panoskaltsis-Mortari

**Affiliations:** 1 Medical Scientist Training Program, University of Minnesota—Twin Cities, 420 Delaware Street SE, Minneapolis, MN 55455, United States of America; 2 Department of Pediatrics, Division of Blood and Marrow Transplant & Cellular Therapy, University of Minnesota—Twin Cities, MMC 366, 420 Delaware St SE, Minneapolis, MN 55455, United States of America

**Keywords:** 3D bioprinting, tissue engineering, esophagus, gastroesophageal junction, alignment, smooth muscle

## Abstract

Engineering functional smooth muscle tissues requires precise control of cellular alignment, particularly in complex anatomical regions such as the gastroesophageal junction (GEJ). We present a scalable wet-spinning approach for generating pre-aligned microtissues (PAMs) from immortalized human esophageal smooth muscle cells embedded in a collagen-alginate core-shell fiber. After maturation, fibers were sectioned into uniform PAMs with preserved alignment and high cell viability. Immunofluorescence and gene expression analyses confirmed the expression of key contractile markers. PAMs were incorporated into a gelatin-methacryloyl bioink and 3D bioprinted to demonstrate alignment along the extrusion path. This method does not require specialized culture platforms and enables efficient production of aligned microtissues for bioprinting. It offers a promising strategy for fabricating anisotropic tissues and may facilitate the reconstruction of complex muscle structures such as the GEJ.

**Abbreviations:**
GEJ: Gastroesophageal junction
GEJHPZ: Gastroesophageal junction high-pressure zone
LECS: Lower esophageal circular sphincter
UGS: Upper gastric sphincter
GEFV: Gastroesophageal flap valve
EMR: Endoscopic mucosal resection
PAM: Pre-aligned microtissue

## 1. Introduction

Muscles, tendons, and other mechanically active tissues have structural elements that allow for efficient generation of, or resistance to, mechanical forces. These structures can be intracellular, such as the cytoskeletal alignment of muscle cells [1, 2], or extracellular, as found with the collagen fibrils in tendons [3]. In muscle tissue, the alignment of thousands or millions of contractile elements allows microscopic forces to sum together for a striking macroscale effect as the entire muscle shortens. Thus, as tissue engineering advances, an increasing interest has developed in creating methods that can recreate the anisotropy found in these tissues. Earlier approaches resulted in the uniaxial alignment of cells by substrate patterning [4], active stretching [5], or mechanical boundary constraints resulting in stress-induced alignment [6]. Combining the principles learned from these studies with new techniques in 3D bioprinting has increased the complexity of the alignment that might be achieved. Extrusion bioprinting, in which a hydrogel bioink is deposited through a nozzle, has been particularly promising for this purpose and has yielded two possible approaches. In one, the macromolecules that make up the hydrogel ink are directed into alignment by controlled material flow during the deposition, guiding cell alignment in the direction of the macromolecular pattern [7]. Another promising approach uses anisotropic microtissues, which are directed into alignment by flow within the extrusion nozzle [8].

A spectrum of complexity is found in these muscle tissues, ranging from simple, unidirectionally aligned cells in many skeletal muscles, to complex, wrapping fascicular structures of the cardiac muscle. One example of a highly complex structure amenable to further tissue engineering efforts is the GEJ, which plays a critical role in disease prevention due to its function as a valve preventing the reflux of caustic stomach contents upwards into the esophagus. Failure of this function can lead to gastroesophageal reflux disease (GERD) and esophageal cancer [9]. If surgical resection is necessary, reconstruction is generally performed by gastric pull-up, which destroys the normal GEJ structure and results in high postoperative morbidity. A tissue-engineered solution to reconstitute the GEJ anatomy could greatly decrease morbidity related to this surgery and could provide additional benefits to the treatment of refractory GERD and other diseases of the lower esophagus. From an engineering perspective, the alignment of the smooth muscle cells within the muscularis externa presents a particular challenge when compared to the rest of the alimentary tract. The two-layered esophageal muscularis with an inner circular layer and outer longitudinal layers transition into the three-layered stomach muscularis with an inner oblique, middle circular, and outer longitudinal layer. This transition gives rise to three important components of the high-pressure zone responsible for tight closure of the GEJ, which are the LECS, the gastric sling fibers, and the gastric clasp fibers [10]. The orientations of the cells within these bundles are not neatly arranged in a simple coordinate system. Replicating this complex structure presents a challenge for current tissue engineering technology [11]. This could possibly be addressed by 3D bioprinting of PAMs which could facilitate faster and more directed alignment compared to biofabrication with single cell suspensions.

**Figure 1.**
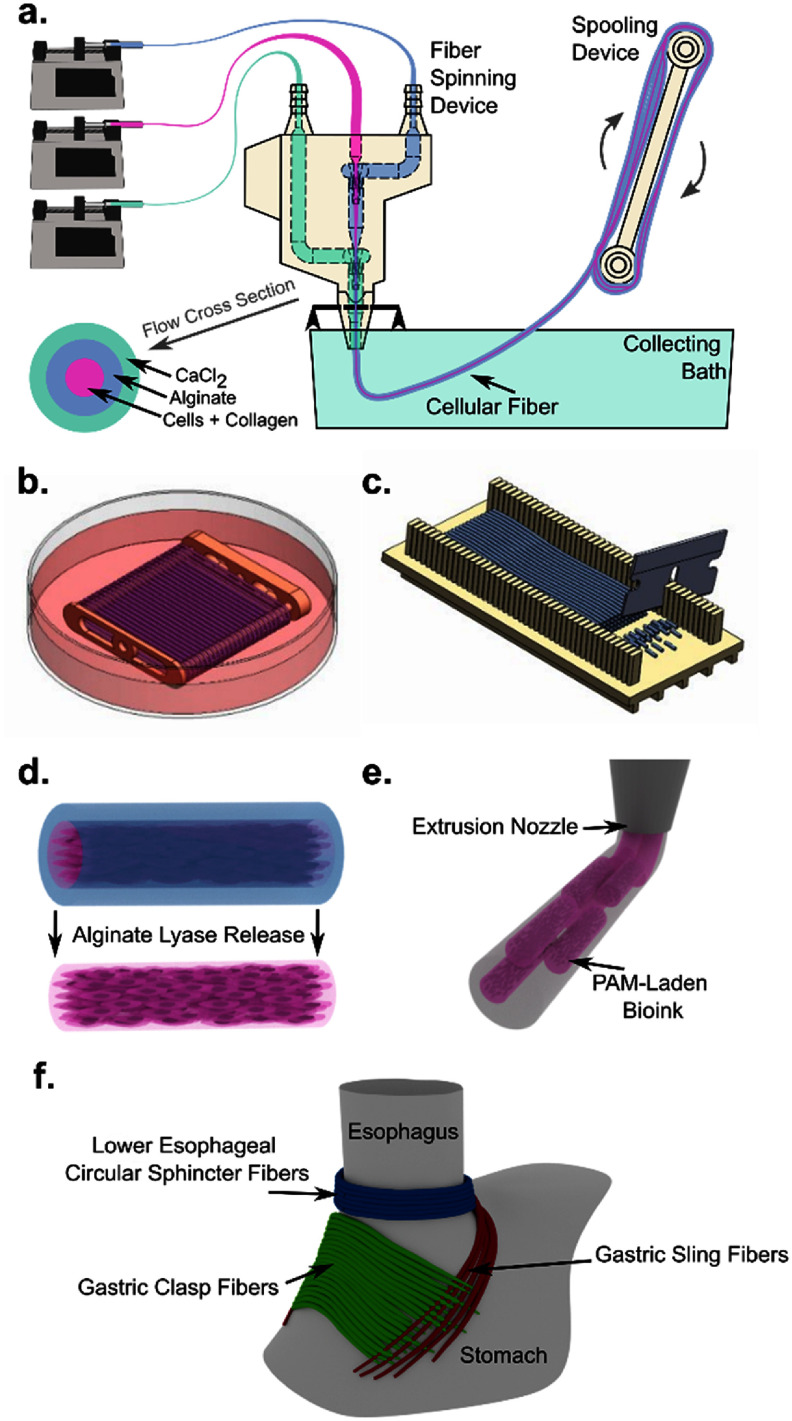
Method for production and bioprinting of PAMs (a) cellular collagen solution, alginate solution, and CaCl_2_ solution are combined in a spinning device to form a solid core-shell fiber, which is wound on a motorized spool. (b) The spool is detached from the motor and the immature fiber is cultured in a dish to form an aligned cellular fiber. (c) The mature fiber is removed from the spool and laid in a cutting device, where it is cut into consistent fragments. (d) Alginate lyase removes the shell from the fragments, releasing the PAMs. (e) PAMs are combined with hydrogel bioink and extruded to lay down the PAMs in alignment with the printing direction. (f) A simplified depiction of the complexity of smooth muscle fascicle alignment at the GEJ, highlighting the non-orthogonal nature of the alignment of the key fascicle groups required for normal valve function.

Compared to previously published alignment methods using bioprinted microtissues, this work presents a novel approach to produce tissues using PAMs created by wet spinning a fiber of hESMCs. Previous approaches to this bioprinting method required the production and use of special culture chambers with microscopic wells in which microtissues were molded [8]. After a period of maturation, dog bone-shaped tissues could be harvested from these microwells [8]. In our work, cells are formed into a single, long, pre-aligned fiber through a wet spinning process (figure 1(a)). Simple, 3D-printed tools are developed to produce the fiber and handle the large amount of tissue that is produced without requiring any special culture chambers. The fiber is then cut into short (2 or 4 mm) fragments that are combined with a hydrogel bioink (figures 1(c)–(e)). Shear-induced alignment of the microtissues through a conical nozzle during 3D extrusion bioprinting results in direct writing of complex muscular tissue alignment. It is expected that this novel approach, in combination with non-planar bioprinting, could address the challenge of reconstructing the GEJ (figure 1(f)) and other complex muscle-based anatomy.

## 2. Materials and methods

### 2.1. Cell culture

Primary human esophageal smooth muscle cells (hESMCs) were purchased from Cell Biologics (H-6089). These hESMCs were immortalized by transduction with recombinant lentiviruses carrying human telomerase (hTERT), cyclin D1 (CCND1), and a variant cyclin-dependent kinase 4 (CDK4R24C) by Capitol Biosciences (Gaithersburg, MD). All genes were transduced in combination with a TET-ON system. These immortalized cells (ihESMCs) were cultured on gelatin-coated tissue culture plastic at 37 °C and 5% CO2 with high-glucose Dulbecco’s modified Eagle’s medium (DMEM) (Gibco 11 965-092) supplemented by 10% FBS (Genesee Scientific 25-514), 100 *µ*g ml^−1^ Primocin (Invivogen ant-pm-2), and 2 *µ*g ml^−1^ doxycycline hyclate (Sigma-Aldrich D9891). Doxycycline was only added during cell expansion culture, and not during fiber production or maturation steps. Cell harvest and passage were accomplished by incubation with TryPLE (Gibco 12563011). Experiments were performed with cells at passages 5–10. Mycoplasma testing was performed at 6 month intervals and found to be negative.

### 2.2. Microfluidic spinning device

Microfluidic devices were created by 3D printing with a Form 3B SLA printer using Surgical Guide Resin v1 (Formlabs RS-F2-SGAM-01). The design featured three fluid channels which converged into two spinnerets in series. Fluids from channels 1 and 2 flowed together at spinneret 1 to form a core/shell flow with fluid from channel 1 in the core. This flow was focused down into spinneret 2, which injected it into the fluid flow from channel 3. The spinneret features were designed with a 600 *µ*m inner diameter and 200 *µ*m wall thickness and were centered in an outer channel with a diameter of 2 mm. Slicing was accomplished using PreForm (v3.28.1) with a layer height of 50 *µ*m. Post-curing was performed in a Form Cure device (Formlabs) at 60 °C for 30 min. Devices were sterilized by autoclaving at 121 °C. To measure the internal dimensions of the finished device, three samples were examined by an XT H 225 (Nikon) *µ*CT scanner and reconstructed with CT Pro 3D XT (v3.1.11, Nikon) software. Segmentation of the resultant files was performed in 3D Slicer (v5.2.2) [12, 13], and diameter measurements were performed in FIJI. Visual mapping of printed component deviation from the computer model of the devices was performed with CloudCompare (v2.12.4) [14].

### 2.3. Microtissue production

Smooth muscle PAMs were prepared by the inclusion of a cellular collagen hydrogel core into a calcium alginate fiber (figure 1(a)). The microfluidic spinning device was attached by flexible tubing to syringes containing core, shell, and sheath solutions [15]. The sheath solution for crosslinking the alginate consisted of 100 mM CaCl_2_ and 3% (w/v) sucrose. The shell solution contained 1.5% (w/v) sodium alginate (Wako Chemicals, 194-13321) with or without 100 *µ*g ml^−1^ erioglaucine dye (Sigma-Aldrich 861146-5 G). Both the sheath and shell solutions were sterilized by autoclave. These solutions were loaded into syringes and connected to the microfluidic spinning device and the fluid lines were primed prior to beginning production of the core solution. The core solution was prepared by mixing sterile bovine collagen (TeloCol 10, Advanced Biomatrix, 5226) with a neutralizing buffer to yield a pre-gel solution at the desired concentration. This was immediately mixed with a cell pellet containing the desired number of cells. This solution was gently pipetted to mix the cells, then loaded into a syringe and immediately put into an ice water-cooled jacket mounted on a syringe pump to prevent premature gelation. The core was connected to the spinning device and the tubing was manually primed.

The tip of the spinning device was placed just below the surface of a collecting bath containing 10 mM CaCl_2_ and 290 mM NaCl in double distilled water. The syringe pumps were engaged sequentially, starting with the alginate shell. Once the shell solution reached the spinneret, the sheath solution was started. When the alginate fiber production was stable, the core solution was introduced. As the fiber was produced, it was spooled using a custom-built motorized spooling device (figure 1(a)). The fiber was collected on a sterile 2-pronged spool that was detached from the device after the spinning process and the collected fiber was placed in petri dishes and covered with culture medium (figure 1(b)). The fibers were incubated at 37 °C and 5% CO_2_ for tissue development. Media was replaced every 2 d. For smaller cultures containing loose, unspooled fibers, aspiration of spent media was performed through a 40 *µ*m cell strainer to prevent accidental loss.

Once the cells had formed long strands of aligned tissue, the fiber spool was transferred to a dish with a sterilized microscope slide. The fibers were released from the spool with two cuts along the ends of the spool. The microscope slides were transferred to a sterilized cutting guide, which was 3D printed using FormLabs Surgical Guide resin (figure 1(c)). This guide allows a razor to pass through every 2 mm. Sterilized single-edge razor blades were used to cut the fibers at the desired lengths and the resulting fiber fragments were transferred to fresh culture medium in the incubator to recover.

The cut tissue fragments were released from their alginate shells to yield the PAMs (figure 1(d)). Alginate lyase (Sigma-Aldrich, A1603) was prepared and aliquoted as a stock solution of 4 mg ml^−1^ and stored at −20 °C. This was diluted 1:50 in culture media to dissolve the alginate shell over the course of 1 h at 37 °C in the incubator. Prior to and during the shell removal, the fiber fragments were incubated for 1 h in 50 *µ*M Y-27 632 (Cayman Chemical 10005583), a ROCK inhibitor, or 5 *µ*g ml^−1^ cytochalasin D (Sigma 2618), an actin polymerization inhibitor, to temporarily prevent fiber shortening during the printing process.

### 2.4. Unaligned controls

Smooth muscle cells were combined with collagen to serve as 3D unaligned controls for comparison to the aligned cell fibers. Cells were prepared in collagen (the same collagen used for the fiber core), then 10 *µ*l droplets were pipetted into petri dishes. The dishes were incubated at 37 °C for 20 min before adding culture media.

### 2.5. Immunofluorescence microscopy

To observe protein expression, wide-field epifluorescence was captured with an EVOS FL Auto 2 (Thermo Fischer) at days 0, 1, 3, and 7 during fiber development. Samples were fixed for 30 min with 4% paraformaldehyde (PFA) at room temperature, then washed 3X for 5 min with DPBS and used immediately or stored at 4 °C for later analysis. Blocking was performed for 1 h with 10% Horse Serum, 1% BSA. Primary anti-human antibodies were diluted in blocking buffer and incubated overnight as follows: Anti-SM-MHC (Proteintech, 21404-1-AP, rabbit polyclonal, dilution 1:100), anti-Calponin-1 (NeoBiotechnologies, 1264-MSM2-P1ABX, mouse monoclonal, dilution 1:200), anti-CX43 (Invitrogen, 13-8300, mouse monoclonal, dilution 1:100). Fibers were washed 3X for 5 min with DPBS before adding secondary antibodies diluted in 1% BSA and incubated at room temperature for 1 h. Secondary antibodies used were Cy3 conjugated with Cy3 goat-anti-mouse (Jackson ImmunoResearch Laboratories, 715-165-151, dilution 1:500) and goat-anti-rabbit DyLight 488 (Invitrogen, 35552, dilution 1:500). Fibers were then washed 3X for 5 min with DPBS. Some fibers were incubated in iFluor 488-conjugated phalloidin (Cayman Chemical, 20549) at 1:1000 dilution without other antibodies. Stained fibers were then rinsed 3X for 5 min in DPBS before mounting with DAPI mounting media and incubating for 1 h.

To image cell alignment, confocal microscopy was performed with a Nikon A1Rsi laser scanning confocal microscope. The samples were fixed for 45 min with 4% PFA at room temperature, then washed 2X for 5 min with DPBS and stored at 4 °C for analysis. The samples were processed in a modified protocol as described for spheroids [16]. Briefly, samples were washed with a penetrating buffer (20% DMSO, 0.2% Triton X-100, 22.5 mg ml^−1^ glycine) for 30 min at RT, then blocked for 2 h at RT with a blocking buffer (10% DMSO, 10 mg ml^−1^ BSA, 0.2% Triton X-100). All buffers were made in DPBS. A buffer (10 mg ml^−1^ BSA, 0.2% Tween 20) was used to wash the samples 5X for 5 min each before adding iFluor 488-conjugated phalloidin at 1:500 dilution and DAPI (5 *µ*g ml^−1^) diluted in antibody dilution buffer. Antibodies were incubated overnight at 4 °C. Samples were washed 5X in DPBS before imaging.

### 2.6. Gene expression

Quantification of the expression of genes related to ihESMC function was performed using TaqMan assays (Applied Biosystems). First, the alginate shell was dissolved from the fibers using alginate lyase, following the same protocol from the microtissue production. The mass of the fibers was measured, and an appropriate volume of TRIzol reagent (Invitrogen) was added. The fibers were homogenized using a rotor-stator homogenizer, and the solution was stored at −80 °C. RNA extraction was performed with chloroform according to the TRIzol manual, and the RNA was collected and washed using RNeasy (Qiagen) spin columns. cDNA was produced using a SuperScript III kit (Invitrogen) and a thermocycler. The qPCR plates were loaded with cDNA samples and TaqMan assays for *MYH11* (Hs00975796_m1), *CNN1* (Hs00959434_m1), *GJA1* (Hs00748445_s1), and *GAPDH* (Hs02786624_g1). Finally, the quantitative polymerase chain reaction (qPCR) was performed using a LightCycler 96 (Roche). Analysis was performed with the LightCycler software using the ddCt method, where *GAPDH* was used as the reference gene and the day 0 sample was used as the reference sample.

### 2.7. Cell viability

Cell viability in 3D constructs was assessed using LIVE/DEAD (Invitrogen, L3224) staining. Cell fibers (day 7 of incubation) were cut using a razor and cutting guide into 4 mm segments. These PAMs were cultured in media for 1.5 h. Dead controls were prepared by incubation in methanol for 30 min. Samples were washed in DPBS (+Ca) for 5 min. Samples for quantification were incubated in ethidium homodimer at 4 *µ*M in DPBS (+Ca) for 30 min at room temperature to label the nuclei of dead cells. Samples for qualitative imaging were also incubated in Calcein AM at 2 *µ*M to label live cells. Epifluorescence imaging was performed immediately after staining. Using FIJI, the average PI signal was measured from the cut tissue ends along the length of the tissue, binning into regions of interest 50 *µ*m along the long axis of the fiber and 200 *µ*m wide. This measurement process was also applied to dead controls, unstained dead controls, and the uncut central region of the living PAMs.

The potential effect of erioglaucine blue dye on ihESMC viability was screened using a CellTite-Glo® 3D (Promega, G9681). ihESMCs were cultured at 10^4^ cells cm^−2^ in gelatin-coated 12-well plates in culture medium with various concentrations of erioglaucine dye for 48 h. Samples were rinsed 3X with 1 ml plain DMEM, then incubated with 0.3 ml DMEM and 0.3 ml CellTiter Glo 3D reagent for 5 min. 200 *µ*l of each sample was transferred to an opaque-walled 96-well plate, and luminescence was measured using a Cytation 3 (BioTek) plate reader.

### 2.8. Bioink formulation

The bioink was made by first diluting glycerol (Sigma-Aldrich, G9012) to 15% (v/v) with double distilled Milli-Q water. Then, lyophilized gelatin methacryloyl (production described previously [17]) was dissolved in the diluted glycerol at 75 mg ml^−1^ at 50 °C for 1 h protected from light. Lithium phenyl-2,4,6-trimethylbenzoylphosphinate (LAP) photoinitiator was dissolved at 10 mg ml^−1^, and 30 mg ml^−1^ xanthan gum was dissolved in this solution to create the hydrogel, which was stirred for 4 h at 37 °C covered and protected from light. The solution was stored at 4 °C until the day of use.

### 2.9. Rheometry

Rheometry was performed on a DHR-3 (Texas Instruments) rotational rheometer with a 2° 40 mm cone and plate geometry on a Peltier heating/cooling base. Collagen working time at 4 °C and gelation time at 21 °C were investigated by oscillatory shear under controlled torque of 10 *µ*N m. Two minutes was allowed for mixing of ice-cold collagen and neutralization buffer and for loading prior to beginning the test. After 30 min of testing at 4 °C, the temperature was increased to 21 °C and the oscillation continued for 30 additional minutes. The gelation point was determined by the crossover of the storage (G’) and loss (G”) moduli.

### 2.10. 3D bioprinting

Fibers were incubated and were cut to length into 2 mm PAMs for bioprinting on day 7. Following treatment with Y-27632 and release from the alginate shells, the PAMs were centrifuged at 300 g for 3 min in 50 ml conical tubes. Due to the high viscosity of the dissolved alginate, the PAMs do not form a pellet immediately. To collect the tissue, half of the solution was aspirated, diluted in media, mixed, and centrifuged until the PAMs formed a pellet at the bottom. The pellet was reconstituted in cell culture media, then transferred into a 5 ml conical and recollected at 300 g for 3 min. After this washing step, the total tissue volume was estimated using the volumetric markings on the conical tube. Taking the measured tissue volume, bioink was added to achieve 30% PAMs by volume at 37 °C. The solutions were mixed using a wide-bore pipette treated with 30 mg ml^−1^ bovine serum albumin to reduce bioink adherence to the side of the pipette tip. This was transferred to a 5 ml gas tight glass syringe (REGENHU, Villaz-St-Pierre, Switzerland) by connecting the pipette tip to the syringe tip, pulling the syringe plunger while simultaneously pushing the pipette plunger in. The PAM-laden bioink was vortexed until it rested against the syringe plunger, and then the syringe was primed to reduce the impact of air in volumetric bioprinting. The PAM-laden Bioink was adjusted to room temperature for bioprinting. A conical syringe tip (18 ga or 20 ga) was added to the glass syringe. The syringe was installed in a R-GEN 200 (REGENHU) volumetric strand dispenser for bioprinting.

Simple, planar constructs were printed on glass slides with a volumetric flow rate of 250 nl s^−1^ with a print speed of 0.24–1.80 mm s^−1^. These parameters were used to automate the production of the construct but were adjusted to ensure smooth extrusion with approximately 1 mm path width. To recreate the structure and alignment of the GEJ, a machine code was created to allow the printer to follow a custom 3D path mimicking the alignment paths of smooth muscle fibers in the anatomy. Due to the non-planar nature of the resulting print paths, a scaffold was needed that would support the print and index to features on the R-GEN 200 build plate. The scaffold was modeled after a GEJ structure and printed on a Form 3B SLA printer using Surgical Guide v1 Resin (Formlabs RS-F2-SGAM-01). Manual control of the print speed was sustained throughout the printing process for the GEJ constructs to allow precise dispensing of bioink along the scaffold. Following printing, constructs were cured for approximately 30 s under 395 nm light, then removed from the scaffold using a pair of forceps and placed in DPBS. These were then fixed in 4% PFA solution for 45 min, washed 3X in DPBS, and then stained with phalloidin 488.

### 2.11. Cell and tissue alignment

Cell fibers and unaligned controls were labeled with DAPI and phalloidin 488 to show the cell alignment using confocal microscopy. Maximum intensity projections of the resultant z-stacks were calculated with FIJI. Image processing of whole images of the phalloidin 488 signal through the OrientationJ plugin produced color-coded images in which the color corresponds to the directionality of the pixels, and saturation corresponds to the coherence. Regional orientation was quantified using the OrientationJ plugin’s distribution function, which was applied to 100 × 100 pixels (62 x 62 *µ*m) square randomly generated regions of interest along the length (*x*-axis) of the fiber or within the whole region (*x* and *y* axes) of images of the unaligned controls. The local window sigma value was 2 pixels, which was used with the cubic spline method in the plugin. Likewise, color-coded images of the printed constructs stained with phalloidin 488 were analyzed but with a sigma value of 10 pixels. These images were captured by the area-scanning mode of the EVOS epifluorescence microscope and were background subtracted prior to color-coding using FIJI’s rolling ball subtraction algorithm and a radius of 100 pixels. Quantification of the alignment of the microtissues within the printed constructs was performed on regions of interest, which consisted of horizontally or vertically oriented sections of the scans, as shown in figure 4(j). The OrientationJ distribution function was used with a sigma value of 10 pixels, and the results from 9 specimens were averaged for both regions of interest.

### 2.12. Statistical analysis

All statistical tests were performed in JMP v15 (SAS). Where appropriate in comparison to a known value, two-tailed *t*-tests were used. Any cases of multiple comparisons were analyzed by ANOVA with post-hoc comparisons using Tukey’s honest significant difference test. Linear regressions were performed as least squares regressions with an alpha of 0.05 in a stepwise process for the elimination of non-significant factors. Graphs for publication were produced in GraphPad Prism.

## 3. Results

### 3.1. Spinning device characteristics

**Figure 2.**
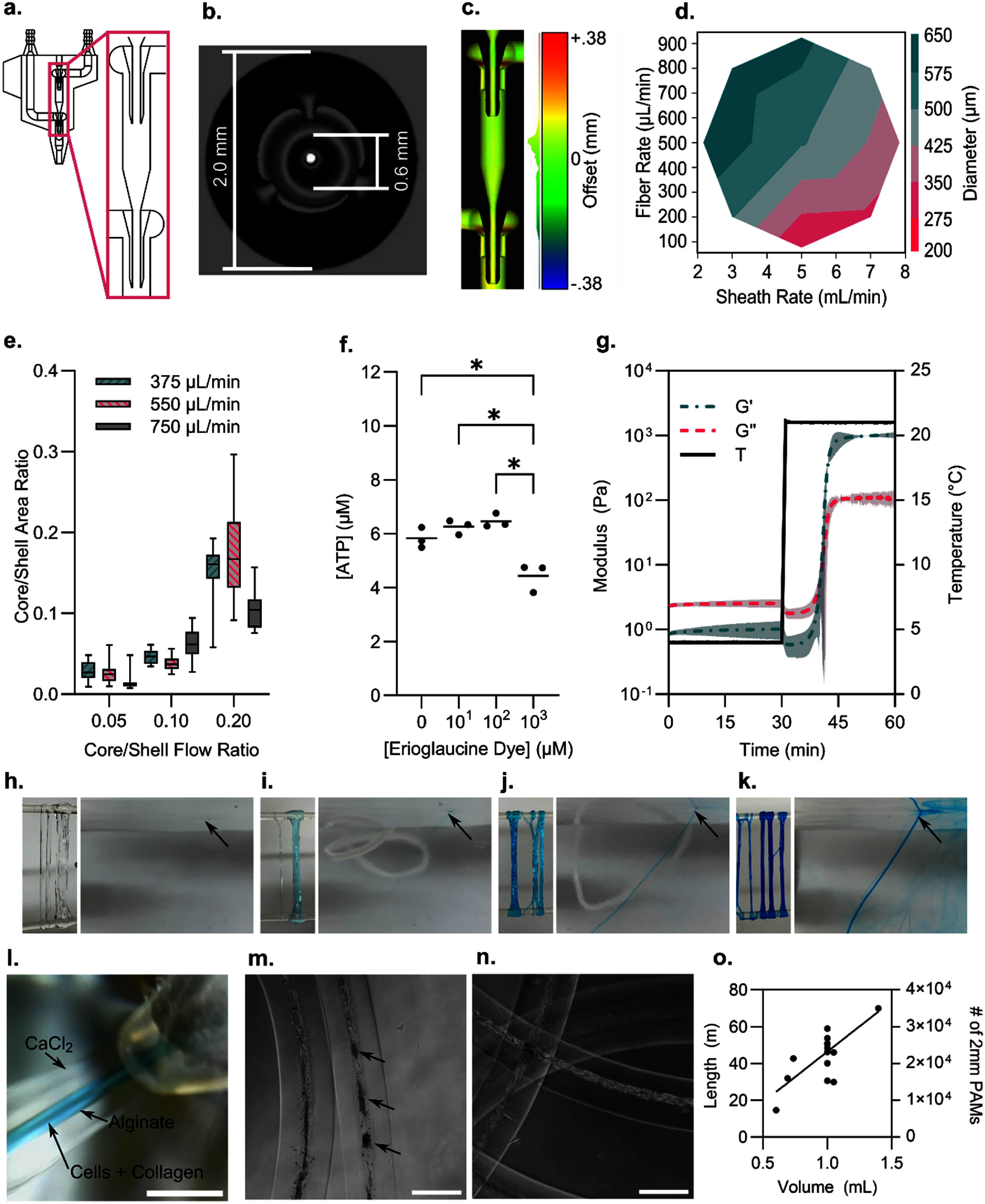
Characteristics of the fiber spinning system. (a) Drawing of the spinning device with the inset image highlighting the two spinnerets in series. (b) A micro-CT scan of the 3D printed spinning device showing one of the internal spinnerets. (c) The micro-CT is compared to the original computer-aided design, demonstrating faithful reproduction of the modeled geometry with interior dimensions slightly narrower than designed, including at key diameters in the spinneret region. (d) The total diameter of the spun fiber increased with the fiber flow rate and decreased with the sheath flow rate. (e) The fiber cross-section depended on the ratio of core and shell flow rates. (f) Erioglaucine dye did not affect measured ATP at or below concentrations of 100 *µ*M (*n* = 3). (g) When maintained at 4 °C, the core collagen solution has at least 30 min of working time for the spinning process and quickly forms a gel when warmed to room temperature. (h)–(k) Adding the erioglaucine dye to the alginate shell greatly increased the visibility of the fiber during the spinning process. Arrows point to the fiber where it can be seen exiting the surface of the crosslinking bath. (h) No dye, (i) 10 *µ*M, (j) 100 *µ*M, (k) 1000 *µ*M. (l) A microscopic view of the submerged tip of the microfluidic device shows the core-shell fiber being carried out by a stream of calcium chloride. (m) Using a cell concentration of 10 million cells per ml, the 4 mg ml^−1^ collagen gel core resulted in an unstable fiber that began breaking into clusters (arrows) by day 7, unlike fibers made with (n) 5 mg ml^−1^ collagen cores which maintained their integrity. (o) The lengths of fiber output and corresponding theoretical numbers of 2 mm microtissues that can be produced from a volumetric input into the core channel of the microfluidic device are shown. For all panels, an asterisk (*) represents *p* < 0.05. Scale bars represent (l) 2 mm and (m, n) 500 *µ*m.

A video of the spinning process is shown in supplemental video 1. The 3D printing process to produce the microfluidic spinning devices successfully created the mesoscale internal geometries required for spinning cellular fibers (figure 2(a)). The internal key diameters are the internal and external diameters around the interior microfluidic spinnerets (figure 2(b)). Semi-quantitative analysis using CloudCompare demonstrated that the micro-CT scans of the microfluidic devices generally conformed to the solid model used to create the printer input, although the interior walls tended to have an offset of 0–0.1 mm, leading to slightly smaller channels than expected (figure 2(c)). The internal diameter of three devices was 454 *µ*m (SD = 68 *µ*m), with an offset of 146 *µ*m from the planned 600 *µ*m (*p* <.0001, *n* = 15). The external diameter was 1920 ± 24 *µ*m, which was 80 *µ*m smaller than the 2000 *µ*m planned diameter (*p* <.0001, *n* = 15).

Flow rates of the three solutions through the device had a predictable effect on the resulting fiber dimensions. Increasing the total fiber flow rate (combination of core and shell flows exiting the distal spinneret) increased the diameter of the produced fiber, while increasing the sheath flow rate led to a diameter decrease (figure 2(d)). An equation to predict total fiber diameter (FD) based on total fiber rate (FR) and sheath rate (SR) was calculated by stepwise regression. The regression model (*F*(2,41) = 42.13, *p* < 0.0001, *R*^2^ = 0.73) was found to be *FD* = 488.95–43.05 *SR +*659.19 *FR*—362.86 *FR*^2^.

Likewise, varying the core and shell components had a predictable effect on the composition of the fiber. Three different core/shell ratios were tested at three different fiber/sheath ratios. The core and shell diameters were measured with FIJI on phase contrast images of the fibers. The diameters were converted to area measurements assuming a circular fiber cross-section. As a simple approximation, it was expected that the area ratio (AR) would be proportional to the flow ratio (FR) between the two convergent streams (figure 2(e)). A linear model of this form was found to be significant (*F*(3,140) = 371.43, *p* < 0.0001, *R*^2^ = 0.72). The resulting equation was *AR* = 0.82 *FR*—0.03.

### 3.2. Fiber shell and core properties

The cellular fibers produced by the spinning process were difficult to see while in solution. For the purposes of demonstrating the technique, the addition of erioglaucine disodium salt (Blue #1) was found to improve visibility, which increased the ease of handling during the spinning process. Visibility increased with increasing dye concentration (figures 2(h)–(k)). Concentrations up to 100 *µ*M did not significantly affect the total concentration of ATP in cell culture compared to no-dye control, but 1000 *µ*M caused a significant decrease (figure 2(f)). This indicates that cell viability and metabolism were unlikely to be affected by lower concentrations of erioglaucine.

The collagen solution used to make the core was tested for working time and viscoelastic mechanics. It was found that a neutralized 5 mg ml^−1^ solution could be maintained in a liquid state for at least 30 min at 4 °C (figure 2(g)). After this working time, the gelation time was found to be 9.3 min (SD = 0.5 min). After 20 min, the G’ was 997.3 Pa (SD = 25.4 Pa), and G’ was 108.2 Pa (SD = 8.4 Pa).

### 3.3. Fiber spinning parameters

Based on the previous results, the typical flow rate for all subsequent experiments was set to 50 *µ*l min^−1^ core, 500 *µ*l min^−1^ shell, and 5 ml min^−1^ sheath. The effects of collagen and cell concentrations were investigated at cell concentrations of 1, 10, 30, and 100 million cells added to 1 ml of 4, 5, or 6 mg ml^−1^ collagen gel (Supplemental figure 1). Collagen concentrations below 5 mg ml^−1^ resulted in instability and collapse of the fiber into cell clusters contained within the alginate shell (figures 2(m) and (n)). The typical concentrations for all subsequent experiments were set to 30 million cells: 1 ml of 5 mg ml^−1^ collagen. It was found that a fiber run could be conveniently planned using 1 ml of core, 10 ml of alginate shell, and 100 ml of sheath solution. The longest recorded continuous fiber was 70 m (estimated from the rotation count of the spool), but the typical production runs resulted in approximately 20–40 m of fiber (figure 2(o)). Linear regression of the input volumes (*V*) and output fiber lengths (*L*) resulted in the following equation*: L =*54.9 *V–*8.5 (*R*^2^ = 0.54, *p* < 0.01).

### 3.4. Morphology in cellular fibers

**Figure 3.**
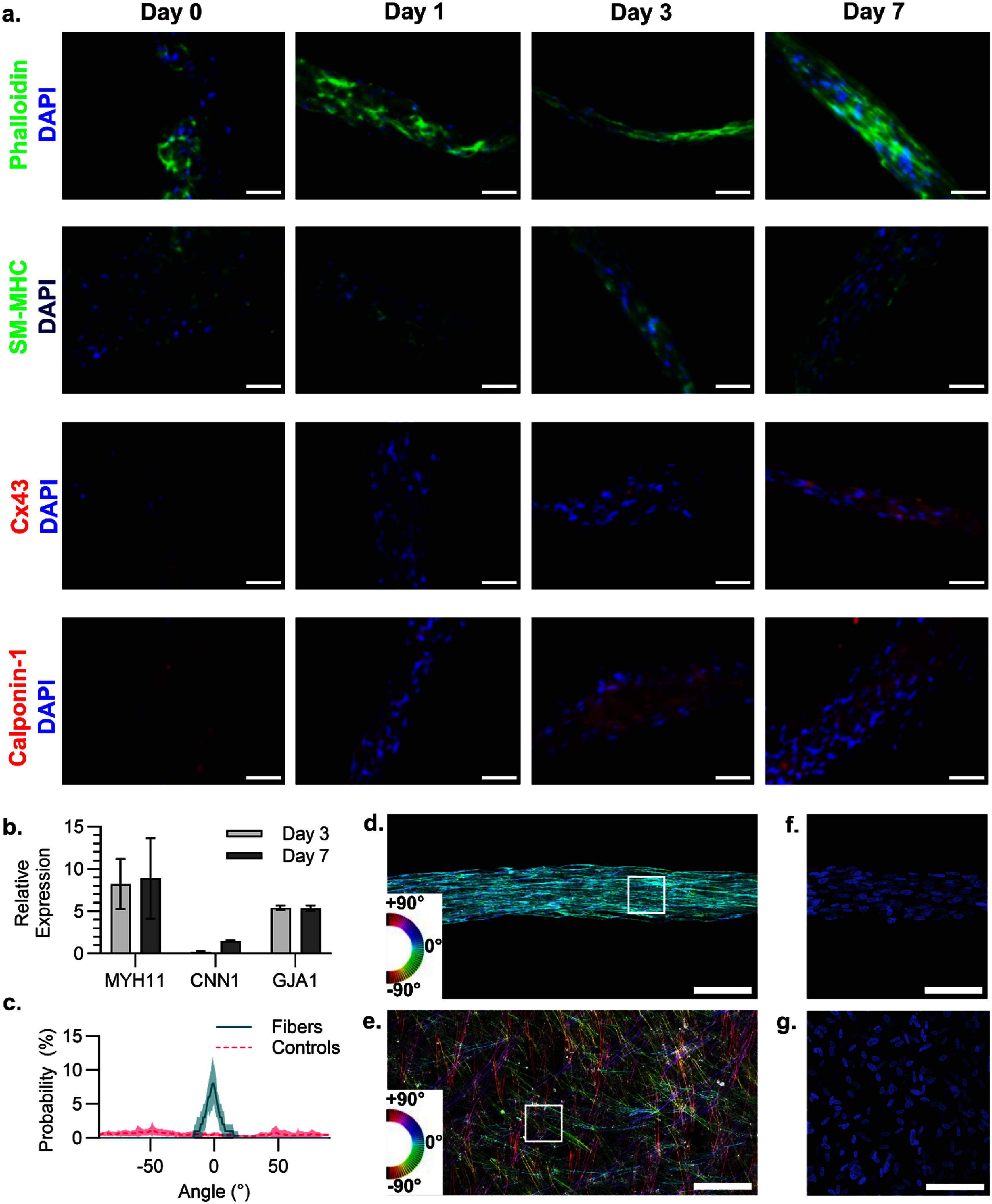
Protein expression and cell alignment during smooth muscle fiber development. (a) Epifluorescence microscopy of the fibers for key markers of smooth muscle cells (SM-MHC, Cx43, and calponin-1) with nuclear counterstains. (b) The normalized expression of the genes (MYH11, CNN1, GJA1) encoding the markers in panel (a) was measured by qPCR and normalized to day 0 expression (*n* = 4). (c) Quantification of the cell alignment within fibers and unaligned controls is shown as a probability density plot, with data extracted from confocal microscopy of phalloidin 488 on day 7 (representative images in panels (d)–(e), *n* = 10 regions of interest, error bars depicted as shaded region show standard deviation). False-colored phalloidin 488 labeling shows the alignment of actin fibers with color-coded directionality, demonstrating cytoskeletal alignment in the (d) aligned fibers but not in the (e) unaligned control. Color-matched legends are shown in the lower left corners of panels (d)–(e), and representative regions of interest used for the quantification in (c) are plotted as white boxes. Scale bars = 100 *µ*m for all panels.

Over the course of 7 d, the fibers developed from loosely associated and unorganized cells within the collagen core into compact tissue, as demonstrated by the phalloidin 488 staining of the actin cytoskeleton (figure 3(a)). Positive staining for SM-MHC confirms the continued expression of a key protein in the smooth muscle contractile unit. Connexin 43, a gap junction protein, and calponin-1, a regulator of smooth muscle contraction, were noted to increase in expression as the tissue developed (figure 3(a)). Quantification of the genes *MYH11* (encoding SM-MHC), *GJA1* (encoding gap junction protein 1), and *CNN1* (encoding calponin-1) was performed with qPCR and is shown in figure 3(b).

Alignment of the cells within the tissue after 7 d was measured by cytoskeletal alignment. Maximum intensity projections of the fiber phalloidin 488 and DAPI signals (figures 3(d) and (f)) demonstrate a high degree of alignment of the nuclei and the actin cytoskeleton compared to the unaligned control (figures 3(e) and (g)). This is especially evident in the phalloidin 488 staining, where the color-coded images of the fiber are largely monochromatic compared to the full spectrum visible in the unaligned control. Quantification of randomly selected regions of interest (boxes in figures 3(d) and (e)) were averaged, and the mean and standard deviation (n = 10) are plotted in figure 3(c). The primary axis set at zero degrees is taken as the long axis of the cell fiber, and the cells are strongly aligned along this axis in the fiber samples.

### 3.5. Effect of cutting on PAMs

**Figure 4.**
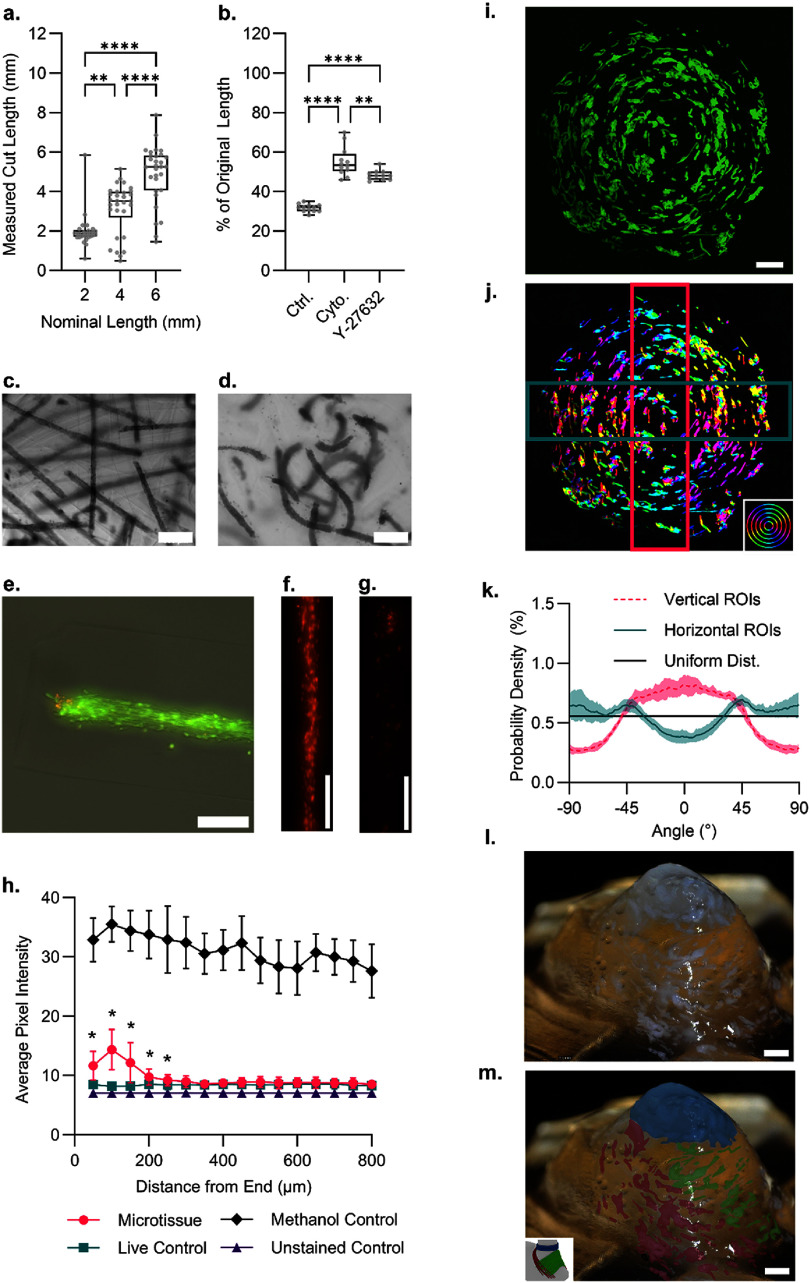
Properties of the PAMs after processing. (a) The cutting guide with a razor produces fibers of different lengths depending on the nominal cutting intervals (*n* = 30). (b) After cutting, releasing the PAMs from the alginate shell results in contraction, which can be partially ameliorated by cytochalasin D (actin polymerization inhibitor) or Y-27 632 (ROCK inhibitor) (*n* = 11–12). Representative images of (c) PAMS before release and (d) Y-27 632-treated PAMs after release. (e) Qualitative imaging with a combination of brightfield, ethidium homodimer (red), and calcein AM (green) of PAM before release from the alginate shell shows cell death at the cut end. Representative images of (f) methanol-treated dead control and (g) cut fiber end (cut end at the top of image) were used for quantification. (h) Quantified LIVE-DEAD staining shows that the cutting process results in moderately increased cell death near the cut compared to the center region of the PAMs. (i) PAMs were bioprinted into circular patterns to demonstrate the alignment capability of the printing method. The phalloidin 488 labeled tissues were then processed with orientationJ to produce false-color images (j) where color indicates alignment direction. An inset image in the lower right of panel j shows the orientationJ color-coding applied to a set of perfect circles, showing how panel j would appear if the microtissues were perfectly aligned with the circular print paths (e.g. elements that are oriented horizontally are coded as cyan, vertical elements are coded red, etc.). The red and blue boxes denote the regions of interest used for alignment quantification. (k) Shows the plots of the mean alignments produced (n = 9), with alignment probability as a function of the angle. The standard deviation is plotted as a shaded region around the mean curve. A theoretical uniform distribution is plotted as an example of a purely unaligned distribution. As expected, the orthogonal ROIs produce complementary probability curves aligning generally at 0° or ±90°. (l) A photograph showing a GEJ-like construct printed onto a scaffold. The gross alignment of the microtissues can be seen in the printed structure. (m) Manual false coloring was added to the photograph to highlight the microtissues in the three printing regions. The inset image depicts the three key fiber directions found in a normal GEJ. Scale bars represent (c), (d) 500 *µ*m, (e), (f), (g) 200 *µ*m, (i, j) 1 mm, and (l, m) 1 mm. Asterisks represent *p* < 0.05 (*), *p* < 0.01(**), *p* < 0.001 (***) and *p* < 0.0001 (****).

The 3D-printed cutting guide allowed the cell fibers to be cut into nominal lengths of multiples of 2 mm. To demonstrate this, acellular alginate fibers cut to 2-, 4-, and 6-mm nominal lengths were measured and found to have mean lengths of 1.98 mm (95% CI [1.68, 2.29]), 3.16 mm (95% CI [2.68, 3.67]), and 4.91 mm (95% CI [4.35, 5.46]), n = 30 (figure 4(a)).

The inhibition of contraction to preserve PAM length was tested using 2 mm cut PAMs (figures 4(b)–(d)). Once cut and released from the alginate shell, the untreated PAMs quickly contracted to 32% of their original length (*n* = 11). Pre-treatment by cytochalasin D before adding alginate lyase and continued treatment during the lyase incubation produced longer PAMs, only contracting to 55% of their original length. Likewise, Y-27632 reduced the contraction, resulting in 49% preservation of the original length.

When cut, it was found that the cells at the ends of the PAMs had increased uptake of ethidium homodimer (red) localized to the nuclei of the damaged cells. Live cells were labeled with Calcein-AM (green). Qualitative images using this live-dead staining show largely living microtissues with some cell death at the cut ends (figure 4(e)). Representative images of the methanol-treated control and cut ends of the PAMs are shown in figure 4(f) and (g), respectively. Quantifying the fluorescence signal along the length of the tissue revealed that the damage was contained to the first 250 *µ*m from the end of the PAM. (figure 4(h)). Compared to the methanol-treated control, the average ethidium homodimer signal was significantly lower even at the cut ends, indicating substantial cell viability in the PAMs where the cut was made.

### 3.6. 3D bioprinting with PAMs

Three patterns were printed with the RGEN 200 to demonstrate alignment of the PAMs by the printing process. An inside-outward print of concentric circular paths produced a circumferentially aligned pattern (Supplemental Video 2). PFA fixation and phalloidin 488 labeling of the f-actin in the printed constructs immediately after printing revealed the arrangement of the PAMs within the structure (figure 4(i)). OrientationJ color mapping was applied to the phalloidin-labeled images (figure 4(j)). This tool calculates an alignment value for each pixel in an image and can produce a color-coded version of the image to indicate the orientation values at each point. As expected in the circular print, a full spectrum of colors is found as the PAMs follow the circular paths. Analysis of the regions of interest showed that there is strong alignment in the sections of the circular pattern (figure 4(k), *n* = 9), and the Kolmogorov–Smirnov test on the two resultant curves was significant (*p* < 0.0001). However, there are points where aggregates of the PAMs disrupt the tissue alignment. As a first step in attempting to recreate the GEJ, scaled-down versions of the anatomy were printed onto plastic scaffolds (figure 4(l), Supplemental Video 3). Briefly, a GEJ-shaped scaffold was printed using a Form 3B printer and surgical guide resin. Using Blender animation software, paths were drawn onto the GEJ model’s surface to approximate the LECS, gastric clasp fibers, and gastric sling fibers. The coordinate points of these paths were extracted using Python and were converted to g-code, allowing for printing of these non-planar paths onto the surface of the GEJ scaffold. These paths allowed for microtissues to be deposited in the desired alignment along the GEJ surface. As visible in figure 4(l), the upper esophagus portion of the printed GEJ tended to collapse, indicating a need for further improvement of the carrier bioink for this method. Nevertheless, bioprinting with PAMs was successful in creating a construct with complex muscle alignment. To increase visibility, false coloring was manually added to the photograph in figure 4(m). A full-resolution image is available in supplemental figure 2.

## 4. Discussion

Various tissues throughout the body rely on highly ordered cell alignment for proper function, especially muscle tissue, due to the need for force summation of the cells for efficient deformation. One such part of the anatomy is the GEJ, which represents both an unmet clinical need in tissue engineering and a challenging structure to replicate in terms of the alignment of the bundles of smooth muscle, which impart its valve-like function. Despite the growing number of processes by which cells can be uniaxially aligned, the complex structure at the GEJ necessitates an approach capable of producing alignment in multiple non-orthogonal planes. 3D bioprinting of PAMs could be such an approach.

Of all the available 3D bioprinting methods, extrusion bioprinting has proven to be an easily implemented method that balances the need for precision and manufacturing speed. Moderate viscosity and yield stress hydrogel bioinks are forced through a nozzle by gas pressure, plungers, or rotating screws. The width of the extrusion is typically between 100 and 1000 *µ*m. The popularity of this method has led to multiple modifications attempting to guide cell alignment. An increasingly common strategy is to cause macromolecular alignment of collagen fibrils along the print direction [18–20]. This phenomenon has been observed both by increasing shear rate and imposition of an extensional flow regime during deposition [20]. A related approach has used a ‘sewing’-like method to prestress a gelatin-based bioink during the printing process, resulting in a high degree of both macromolecular and cellular alignment [7]. Extension of the concept of printed alignment into the mesoscale domain was demonstrated by extrusion printing of densely packed hydrogel microstrands, which became moderately entangled during the printing process but resulted in overall alignment with the printing direction [21]. Recently, a similar approach to the PAM printing presented here was shown with induced pluripotent stem cell-derived cardiomyocytes. In that study, microtissues were produced by suspension between silicone cantilevers and resulted in dog bone-shaped microtissues, which aligned well with the printing path, producing significant anisotropy in the macroscopic contraction of the printed tissues [8].

The first challenge for implementing the PAM printing method is producing highly aligned microtissues efficiently. While suspension between cantilevers has been used frequently in the past, our experience with this in preliminary studies found it to be a challenging method to scale up (data upon request). Production of meters-long cellular fibers through microfluidic core-shell encapsulation showed that smooth muscle-like cells could align during fiber maturation [15]. In our work, the fiber spinning device was modified to be produced by stereolithography printing, which allowed for rapid iteration of prototypes with low time and cost. The resulting spinning system produced tunable fiber properties based on flow rates. The fiber method lends itself to scaled-up production, as no custom molds are required. Although the longest cellular fiber produced in this work was at least 70 m long, modifications to the system could theoretically allow for significant increases in length and a corresponding increase in the number of PAMs produced. Due to the extreme length of the fibers, a motorized spooling device was created that both increased the ease of handling and maintained the fibers in macroscale alignment for eventual cutting into PAMs. The high degree of cellular alignment within the fibers and the small amount of cell death associated with the cutting process confirms that this approach could be a path toward large-scale production of PAMs for bioprinting. When printed in a photocrosslinkable bioink, the PAMs aligned with the printing direction. An early attempt to extend this alignment into GEJ-like constructs showed that non-planar printing with this method is possible, but more work will be needed to increase the tissue density and improve the alignment. Notably, an expected limitation of this method is that small-scale constructs with short path lengths, frequent path interruptions, and tight turns will decrease the alignment, which is consistent with what was found while printing the small-scale GEJ. In future work, it is expected that combining this printing method with non-planar printing either on a scaffold or in a support bath will facilitate the creation of full-scale GEJ-like constructs.

This new approach to generating microtissues for 3D bioprinting alignment differs from the previously published cardiac microtissue method in a few ways. The previous dog bone-shaped molding approach used combinations of iPSC-derived cardiomyocytes and fibroblasts suspended in a 1 mg ml^−1^ collagen hydrogel supplemented with 1.25 mg ml^−1^ fibrinogen [8]. To harvest from the micropillar arrays, it was necessary to leave the micropillars without a retaining cap. This produces an undesirable failure mode by which the microtissues can slip off of the micropillars by deflecting the pillars inward, which resulted in approximately 50% failure rate after 5 d of development [8]. Thus, tissue harvesting was performed at day 3 of development, where most of the microtissues were still adhered. In our new fiber spinning method, stable fibers can be produced with greater development times. We and other groups have maintained fibers for over a month [15]. This is anticipated to allow for more mature tissue development compared to micropillar molding. This is consistent with our experience with molding approaches, where we found similarly high failure rates over a 6 day period, leading to the development of our fiber spinning approach as an alternative.

The scalability of fiber spinning is greater than that of molding due to decreased raw material input and increased packing density. While the molding approach requires a long double-molding process to make the required plates, wet spinning only requires common syringe pumps and a reusable spinneret device. Improved process efficiency was achieved by including a low-cost spooling device to manage long fiber production. The spooled fiber allows for a 3D packing density that is not possible with molding, since the molds are manufactured as a single layer. The previous molding approach allowed 1050 microwells per well plate, with each tissue being 2 mm in length. In comparison, an equivalent number of microtissues could be produced by 2.1 meters of wet-spun fiber. As the spinning process typically produced approximately 30 m of fiber per production run (15 000 microtissues at 2 mm cut length), which was stored in a 100 × 25 mm petri dish, the typical microtissue density was approximately 76 microtissues per cubic centimeter of culture chamber volume during incubation. Our group has observed that significantly longer cellular fibers (approximately 70 m) can be placed similarly into a petri dish, increasing its packing efficiency. This is compared to an estimated 85.5 × 127.8 × 17.3 mm volume of the molding approach, that yields approximately 6 microtissues per cm^3^. Thus, the manufacturing efficiency in terms of required volume is at least ten times greater with the fiber spinning method. The actual maturation time of the tissues cannot be directly compared between the methods at this time due to the differences in cell types included, but it is expected that the time of incubation to form mature microtissues is comparable between the methods. The production time of a 30 m fiber using 1 ml of core fluid and a rate of 50 *µ*l min^−1^ is 20 min. Although this process step cannot be easily compared to the molding approach, there is a possibility that multiplexing multiple spinning devices together with greater volumes of fluid could lead to significantly higher production rates.

Finally, there is a difference between the molding and spinning methods in the morphology of the microtissue ends. In the molding method, the ring-shaped ends of the tissue disrupt the desired alignment pattern. Alternatively, the fiber spinning approach requires cutting living cells, which causes damage to the tissue at the ends out to 250 *µ*m from the cut. However, substantial numbers of living cells remain even at the cut ends of the tissue. Further studies will be required to compare the fusion behavior of these two microtissue bioprinting approaches during the maturation of the printed tissue constructs.

There are limitations to our study with respect to cellular biology and the bioprinting process. First, the ihESMCs used here, while convenient for their greatly increased expansion potential in 2D culture, will not be appropriate for use as tissue-engineered grafts for *in vivo* use. Future work will ideally utilize induced pluripotent stem cells differentiated to a smooth muscle lineage for histocompatibility with treated patients. Second, increased tissue density, optimization of tissue length, and optimization of bioink parameters are necessary for the ideal implementation of this method. For all discussed methods of bioprinting cell alignment, questions remain as to how robust the alignment will be when exposed to complex forces that may be experienced within a bioreactor or post-implantation into a patient. Whether pre-maturation of microtissue fragments may be beneficial to faster tissue development and preservation of alignment compared to methods based on matrix-based alignment cues remains to be seen.

## 5. Conclusion

In this work, we present a novel process to efficiently produce PAMs for 3D printing applications. Immortalized smooth muscle cells can be formed into aligned tissue by entrapment within a core-shell fiber. This fiber can be cut into fragments, producing microtissues that can be combined with a bioink and extruded by a 3D bioprinter in a manner resulting in the alignment of the microtissues and the cells contained within. This wet-spinning process allows for rapid microtissue production that can be used as material inputs for later engineering processes. We have demonstrated the potential for increased efficiency in production time and culture volume. In future work, this method of producing PAMs will require optimization to allow for 3D bioprinting of the GEJ and other similarly complex tissues.

## Data Availability

The data cannot be made publicly available upon publication because no suitable repository exists for hosting data in this field of study. The data that support the findings of this study are available upon reasonable request from the authors.
